# Supplementary material for: Structural basis of promoter recognition by Staphylococcus aureus RNA polymerase
Source: Nat Commun. 2024 Jun 6;15:4850. doi: 10.1038/s41467-024-49229-6 (PMC11156646; doi:10.1038/s41467-024-49229-6)
Supplement: Supplementary file 1 — Supplementary Information [file 41467_2024_49229_MOESM1_ESM.pdf]

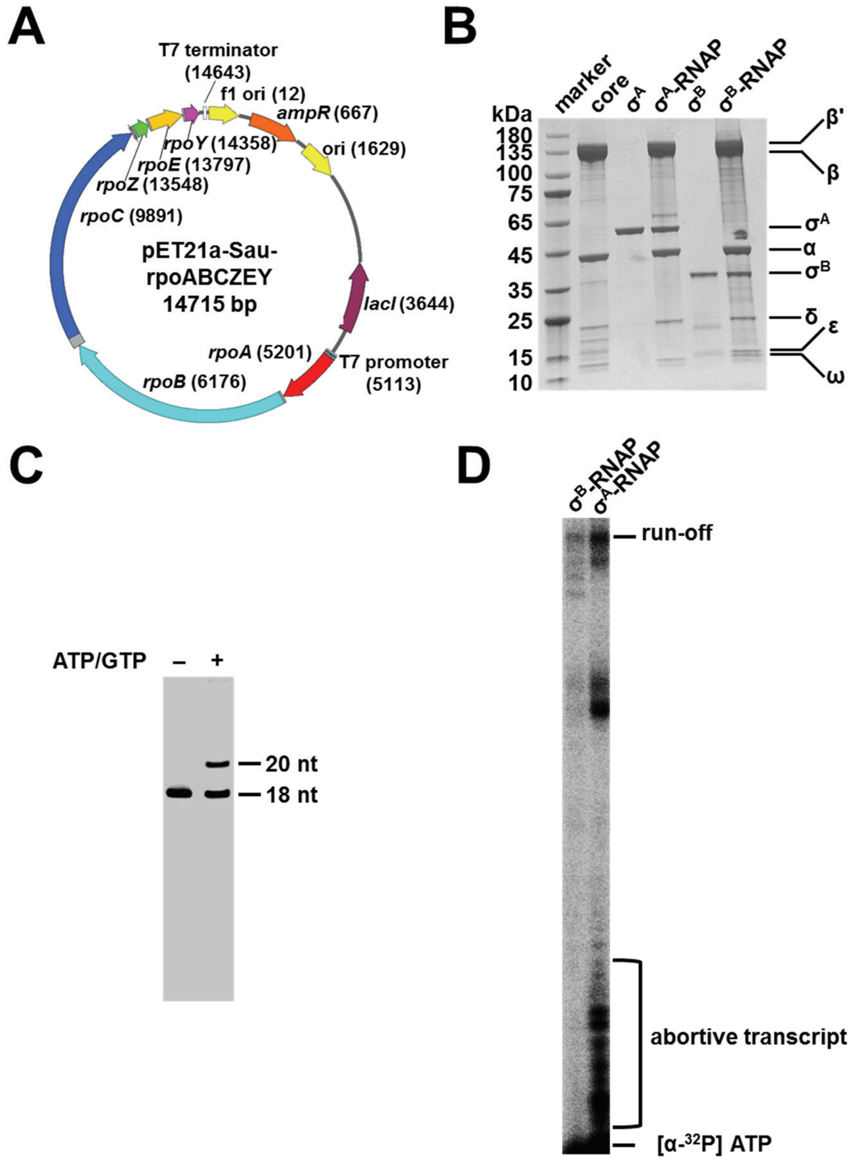

Supplementary Figure 1. Purification of *S. aureus* RNAP.

(A) The map of plasmid pET21a-Sau-rpoABCZEY.

(B) SDS-PAGE of *S. aureus* σ^A, σ^B, RNAP core and holoenzyme. Source data are provided as a Source Data file. Experiments were repeated independently three times with similar results.

(C) Primer extension assay confirms the activity of *S. aureus* RNAP core enzyme. Source data are provided as a Source Data file. Experiments were repeated independently three times with similar results.

(D) Run-off transcription assay confirms the activity of *S. aureus* RNAP holoenzyme. Source data are provided as a Source Data file. Experiments were repeated independently three times with similar results.

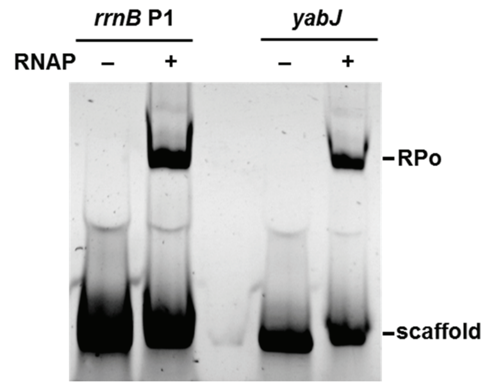

Supplementary Figure 2. EMSA confirms the formation of σ^A-RPo and σ^B-RPo.

1.2 μM DNA scaffold is incubated with or without 1 μM *S. aureus* RNAP holoenzyme for 10 min at 37°C. Source data are provided as a Source Data file. Experiments were repeated independently three times with similar results.

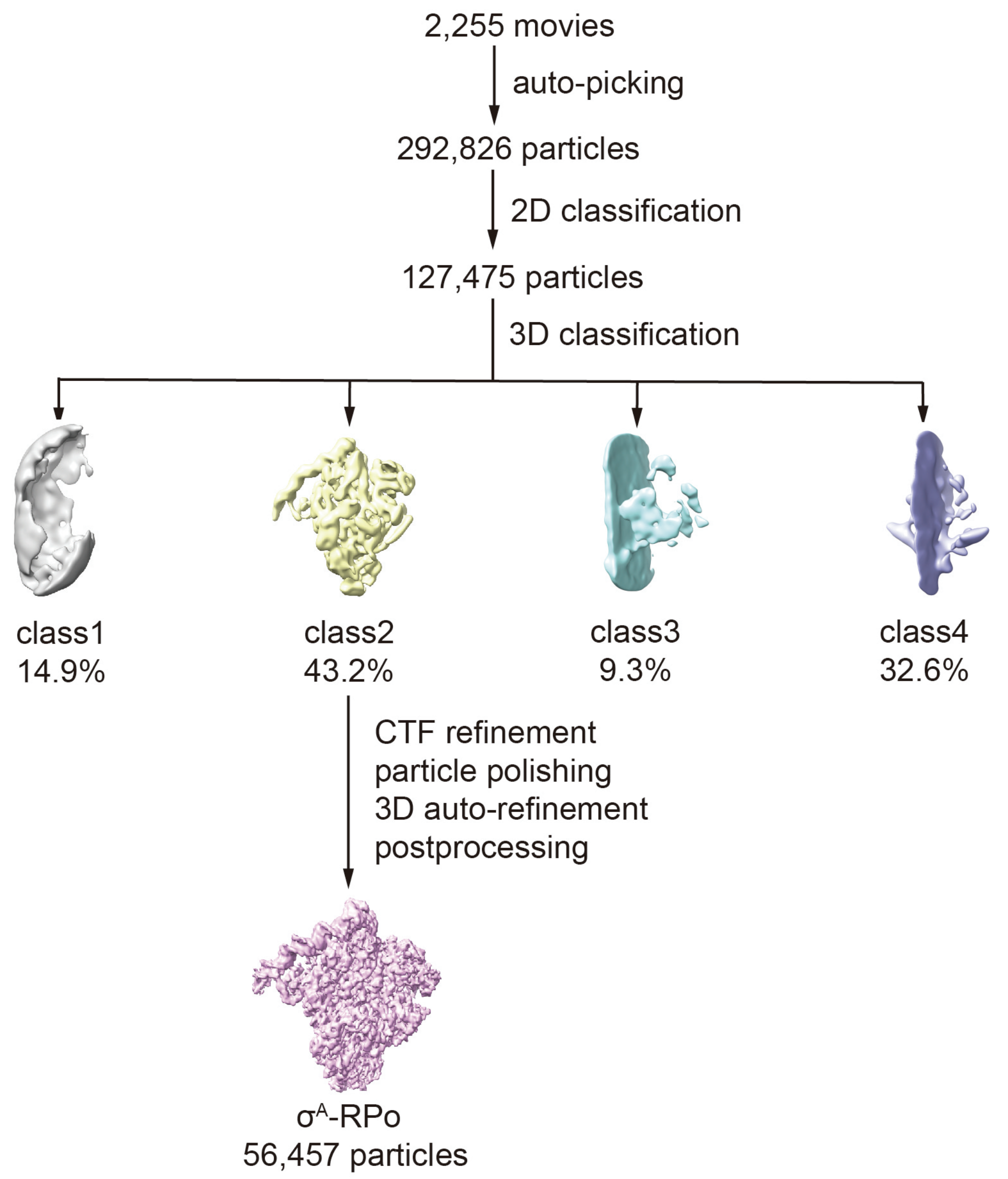

Supplementary Figure 3. Data processing pipeline for σ^A-RPo.

292,826 particles were picked from 2,255 movies using templates in RELION. After 2D classification, 127,475 particles were selected for 3D classification. After 3D classification, 56,457 particles were selected for auto-refinement, CTF refinement, particle polishing, and postprocessing.

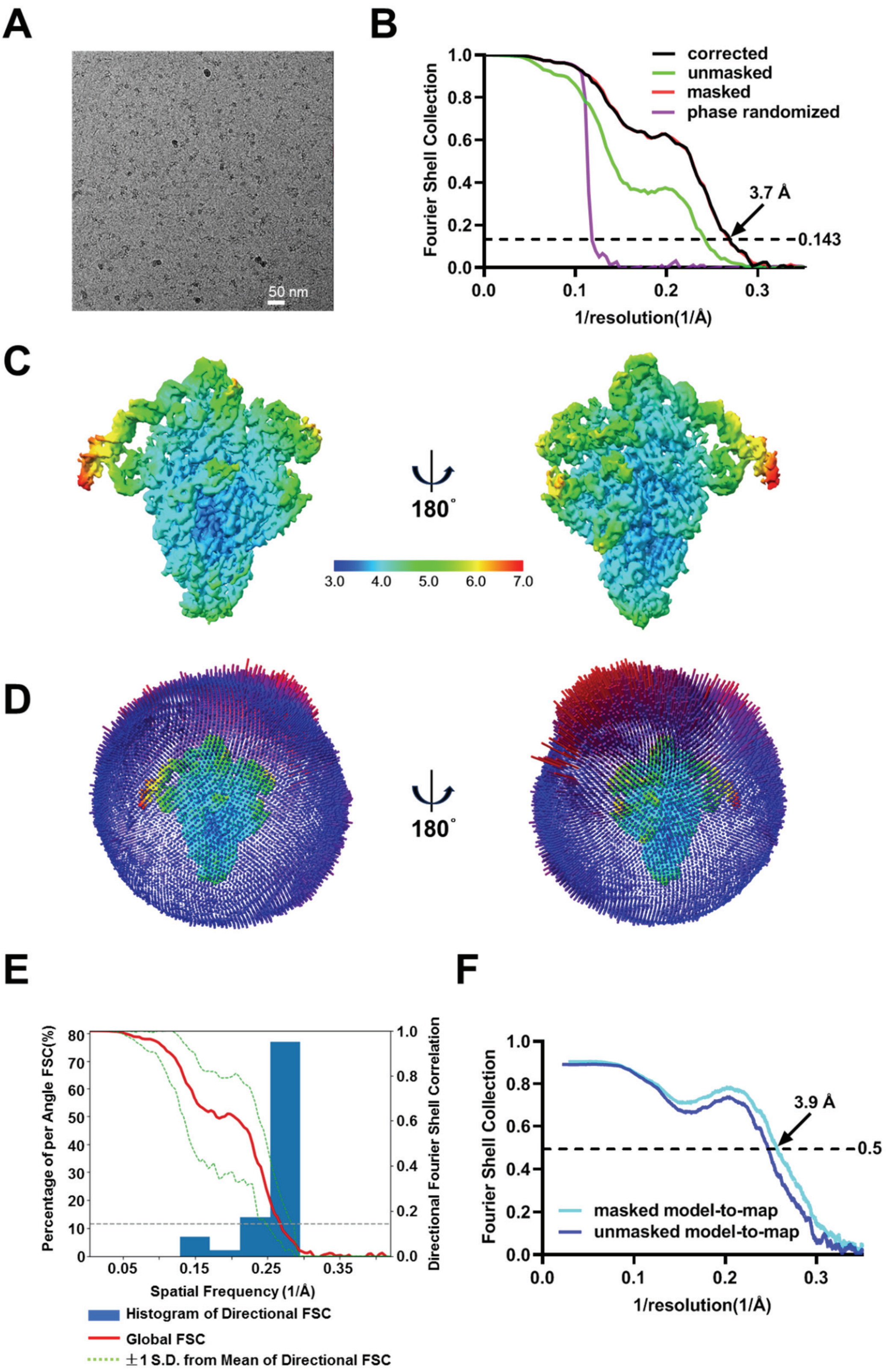

Supplementary Figure 4. Data validation for σ^A-RPo.

(A) A representative cryo-EM micrograph of σ^A-RPo.

(B) Corrected, masked, unmasked, and phase randomized FSC curves. The dashed line

represents the 0.143 FSC cutoff.

(C) Cryo-EM density map colored by local resolution.

(D) Angular distribution of particle projections.

(E) The 3DFSC curve was created on https://3dfsc.salk.edu/.

(F) Masked and unmasked map-to-model FSC curves. The dashed line represents the 0.5 FSC cutoff.

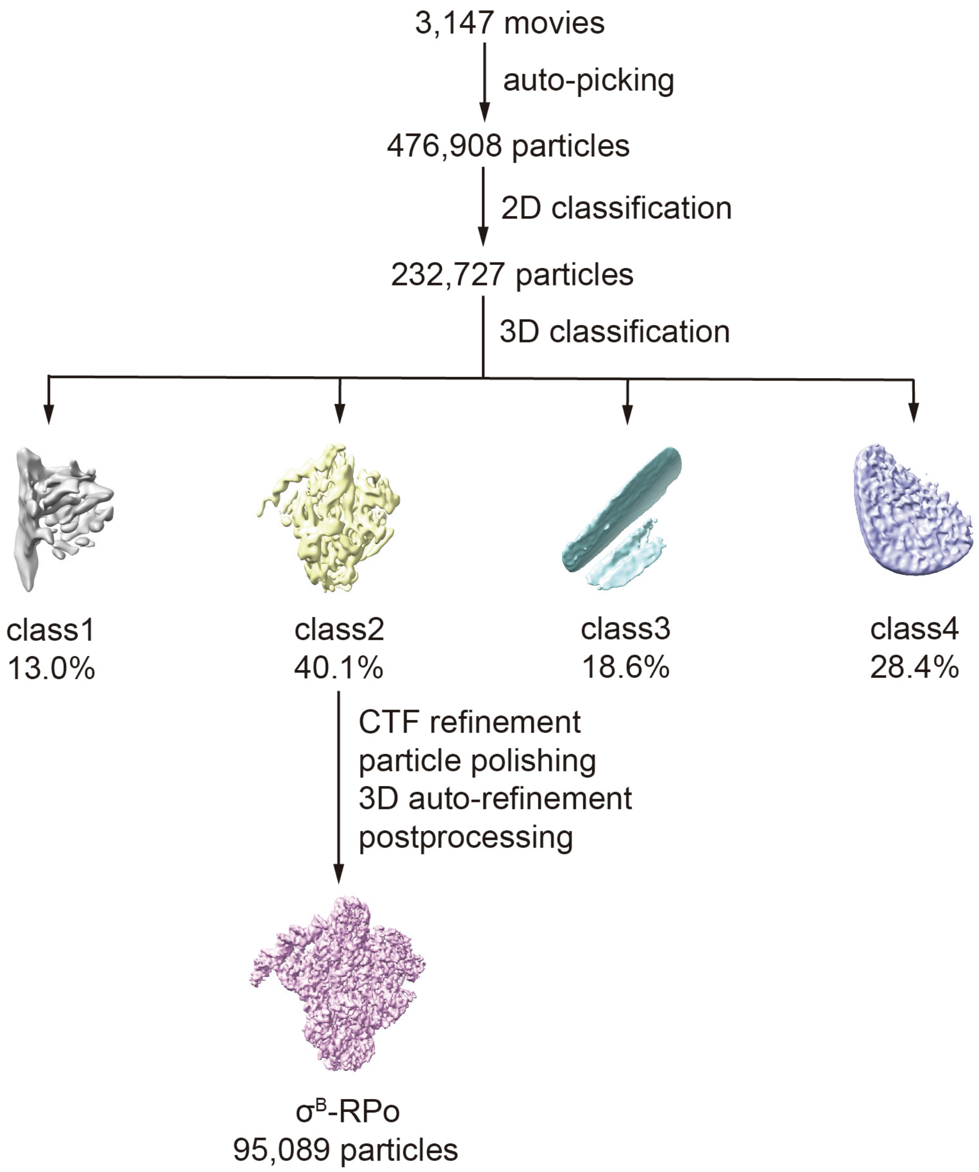

Supplementary Figure 5. Data processing pipeline for σ^B-RPo.

476,908 particles were picked from 3,147 movies using templates in RELION. After 2D classification, 232,727 particles were selected for 3D classification. After 3D classification, 95,089 particles were selected for auto-refinement, CTF refinement, particle polishing, and postprocessing.

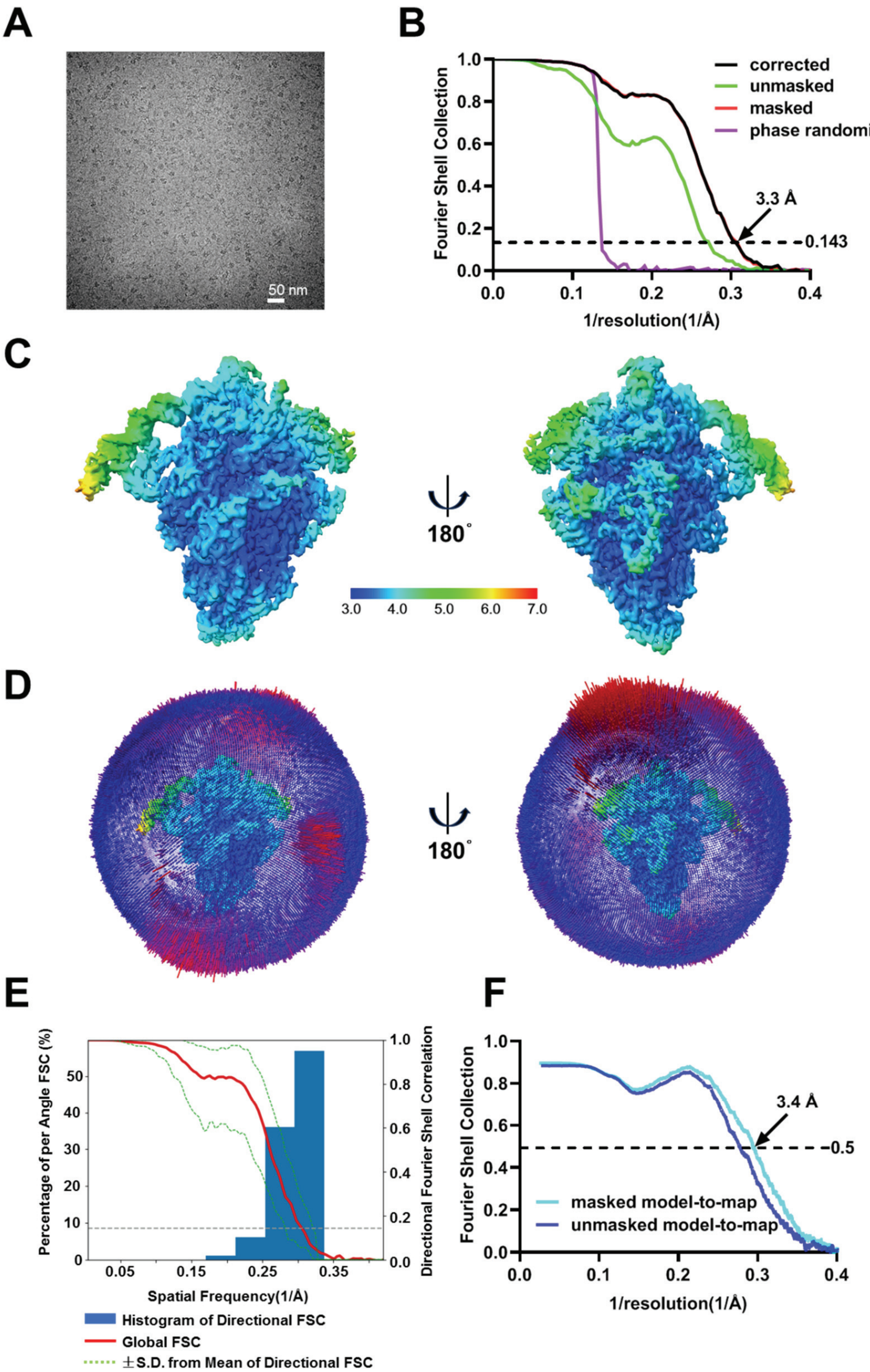

Supplementary Figure 6. Data validation for σ^B-RPo.

(A) A representative cryo-EM micrograph of σ^B-RPo.

(B) Corrected, masked, unmasked, and phase randomized FSC curves. The dashed line represents the 0.143 FSC cutoff.

(C) Cryo-EM density map colored by local resolution.

(D) Angular distribution of particle projections.

(E) The 3DFSC curve was created on https://3dfsc.salk.edu/.

(F) Masked and unmasked map-to-model FSC curves. The dashed line represents the 0.5 FSC cutoff.

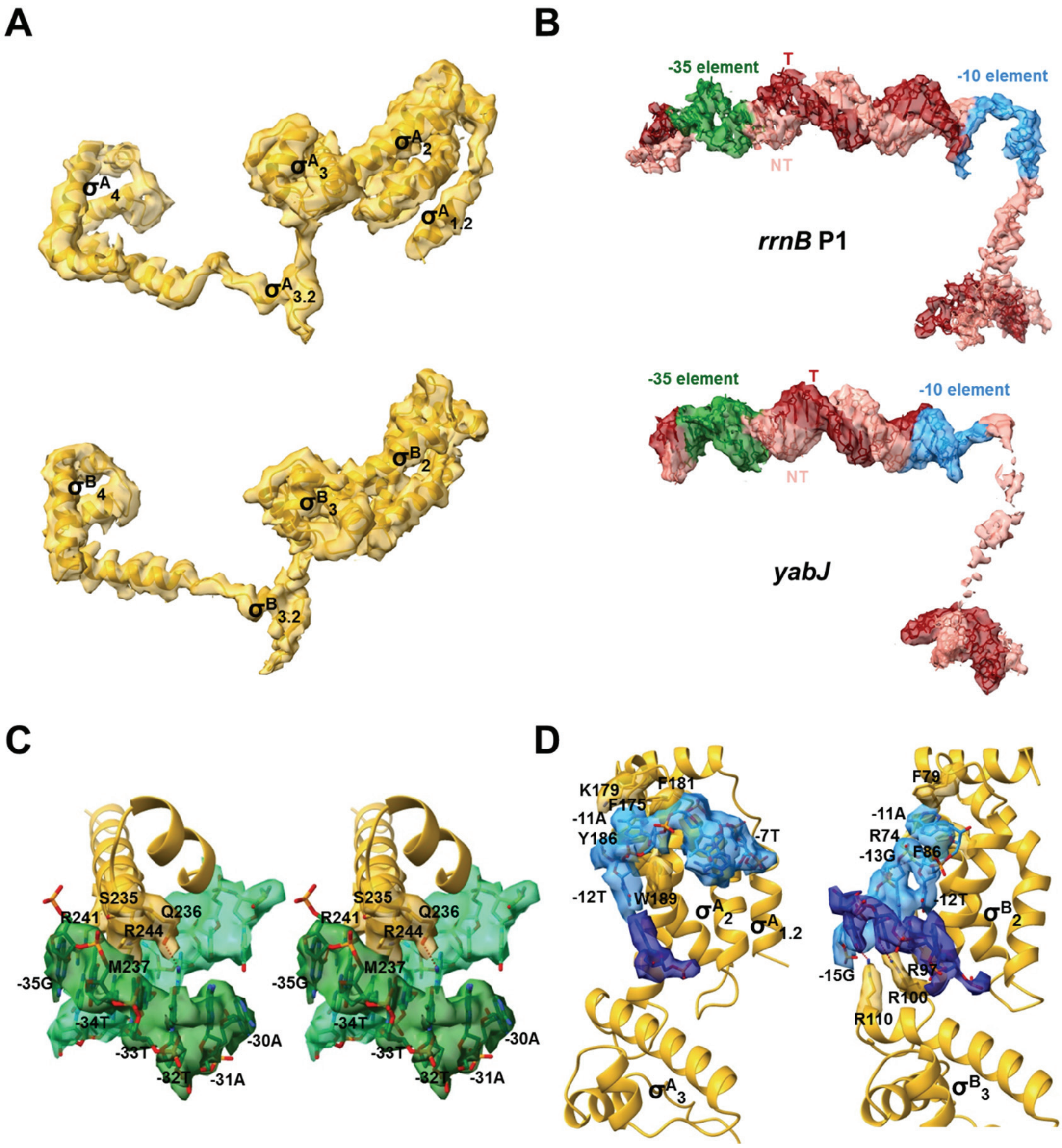

Supplementary Figure 7. Representative electron potential map and superimposed model.

(A) The electron potential map without B-factor sharpening and the superimposed model of σ^A and σ^B. The contour level is 0.01. The carve radius is 3.4 Å.

(B) The electron potential map without B-factor sharpening and the superimposed model of *rrnB* P1 and *yabJ*. The contour level is 0.01. The carve radius is 3.4 Å.

(C) The electron potential map with B-factor sharpening and the superimposed model of σ^B_4-DNA. The contour level is 0.01. The carve radius is 3.4 Å.

(D) The electron potential map with B-factor sharpening and the superimposed model of σ^A-DNA and σ^B-DNA. The contour level is 0.01. The carve radius is 3.4 Å.

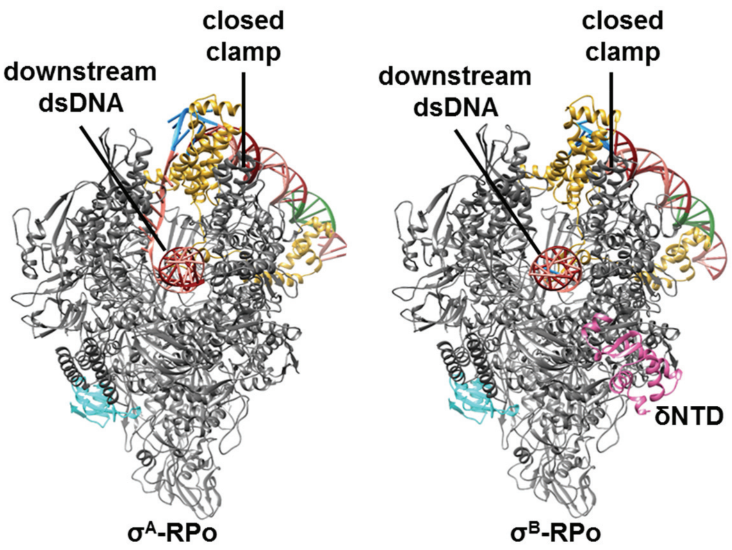

Supplementary Figure 8. The clamp adopts a closed conformation, securing the transcription bubble and downstream dsDNA in the main channel.

The models of σ^A-RPo (left) and σ^B-RPo (right) are shown in cartoon representation. Gray, RNAP core except ε and δ; cyan, ε; pink, δ; yellow, σ^A and σ^B; salmon, nontemplate strand DNA; red, template strand DNA; green, the -35 element; blue, the -10 element.

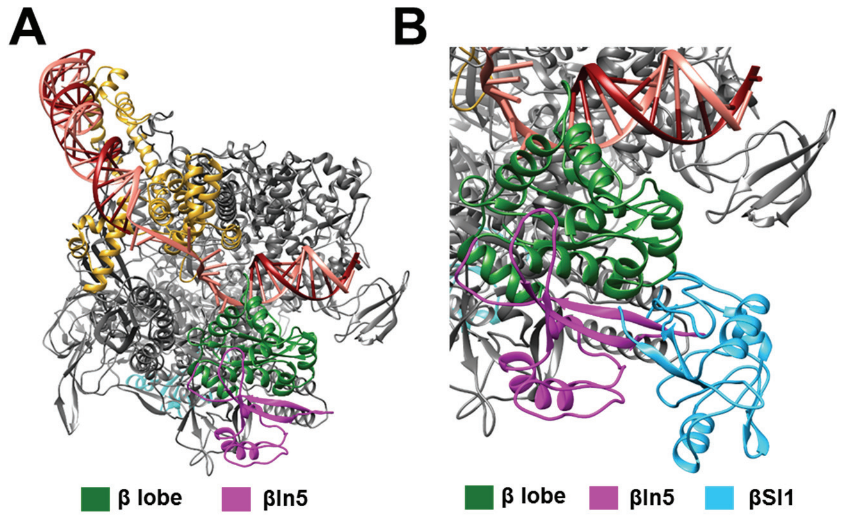

Supplementary Figure 9. βIn5 inserts into and packs against the β lobe.

(A) The overall structure of *S. aureus* σ^A-RPo is shown in cartoon representation. Gray, RNAP core; cyan, ε; magenta, βIn5; green, β lobe; yellow, σ^A; salmon, nontemplate strand DNA; red, template strand DNA.

(B) Structural superimposition of *E. coli* σ^{70}-RPo (PDB: 6CA0) and *S. aureus* σ^A-RPo. Gray, *S. aureus* RNAP core; cyan, *E. coli* βSI1; magenta, *S. aureus* βIn5; green, *S. aureus* β lobe; salmon, nontemplate strand DNA; red, template strand DNA.

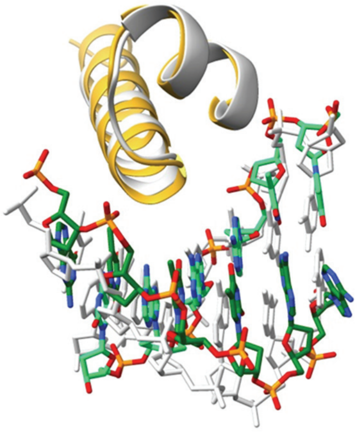

Supplementary Figure 10. σ^B_4 interacts with the -35 element in the same way as σ^A_4. Comparison of σ^A_4-DNA interactions (gray) and σ^B_4-DNA interactions (colors as in Fig. 2A). The models of σ^A_4 and σ^B_4 are shown in cartoon representation. The models of -35 element are shown in sticks.

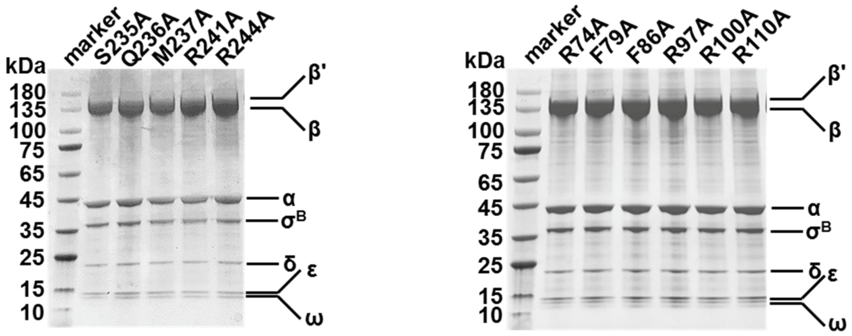

Supplementary Figure 11. σ^B derivatives form holoenzyme with RNAP core.

σ^B derivatives were incubated with RNAP core enzyme. Then size exclusion chromatography and SDS-PAGE were used to confirm that σ^B derivatives can form holoenzyme with RNAP core. Source data are provided as a Source Data file. Experiments were repeated independently three times with similar results.

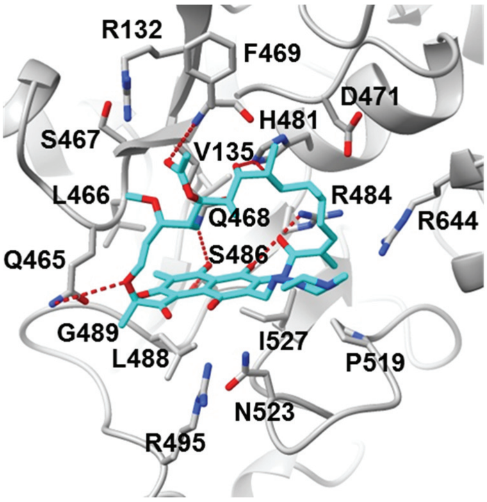

Supplementary Figure 12. Structural modeling of *S. aureus* RNAP-rifampin complex.

S. aureus RNAP is shown in cartoon representation. Rifampin and its interacting residues are shown in sticks. Hydrogen bonds are shown in red dashed lines.

Supplementary Table 1. List of σ^B-dependent promoters.

Gene id	-35 element	length of spacer	-10 element
ccrA	TTTTAA	12	GGGGAT
yabJ	GTTTAA	14	GGGTAT
esxA	GTTTAA	12	GGGTAT
egc	GATTAG	13	GGGTAT
katA	GTTTAA	14	GGGTTA
asp23	GTTTAA	14	GGGTAT
opuD	GATTAA	14	GGGTAT
clpL	GTTTTA	14	TGGAAA
clfA	GATTAA	13	GGGTAT
csb3	GTTTAA	14	GTGTAT
csb4	GTTTAA	14	GGGAAA
csb7	GTGTGA	14	GGGTAG
csb8	GTTTAG	13	GGGTAA
csb9	GTTTTA	14	TGGTAT
csb12	GTTTTA	14	GGGTAA
csb19	GTTTAG	14	CGCTAT
csb24	GTTTAT	14	GGATAA
csb28	GATTAA	15	GGGTAA
csb33	GTTTGA	14	GGGAAT
sa0455	GTTTAA	14	GGGTAT
sa0572	GTATAT	12	GGGAAT
sa0772	GTTTAG	13	GGGTAA
sa0752	GTTTAA	14	GGGTAA
sa2452	GATTCA	13	GGGTAA
sa0632	GTTTTA	13	GGGTAT
sa2309	GTTTAA	14	GGGAAA
sa0359	GAATAA	13	GGGTAA
mw0922	GTTTAA	14	GGGTAT

Supplementary Table 2. Cryo-EM data collection and refinement statistics.

	σ^A-RPo	σ^B-RPo
**Data collection and processing**		
Microscope	Titan Krios	Titan Krios
Voltage (kV)	300	300
Detector	Falcon 4	Falcon 4
Electron exposure (e/Å^2)	51	51
Defocus range (μm)	1.0-2.0	1.0-2.0
Data collection mode	Counting	Counting
Physical pixel size (Å/pixel)	1.19	1.19
Symmetry imposed	C1	C1
Initial particle images	292,826	476,908
Final particle images	56,457	95,089
Map resolution (Å)[a]	3.7	3.3
**Refinement**		
Root-mean-square deviation		
Bond lengths (Å)	0.004	0.003
Bond angles (°)	0.603	0.606
Molprobity statistics		
Clashscore	9	9
Rotamer outliers (%)	0.07	0.18
Cβ outliers (%)	0	0
Ramachandran plot		
Favored (%)	98	98
Outliers (%)	0	0
Map-to-model correlation coefficient	0.82	0.84

[a]Gold-standard FSC 0.143 cutoff criteria.
