# Supplementary material for: Structural basis of promoter recognition by Staphylococcus aureus RNA polymerase
Source: Nat Commun. 2024 Jun 6;15:4850. doi: 10.1038/s41467-024-49229-6 (PMC11156646; doi:10.1038/s41467-024-49229-6)
Supplement: Supplementary file 3 — Description of additional supplementary files [file 41467_2024_49229_MOESM3_ESM.pdf]

Description of Additional Supplementary File

Supplementary Data 1:- The sequences of oligonucleotides and plasmids in this work.
